# Supramolecular Chirogenesis in Porphyrin-Based Systems: Chirality Transfer from Anionic Chiral Surfactants to Cationic, Achiral Porphyrins

**DOI:** 10.3390/ijms262311330

**Published:** 2025-11-24

**Authors:** Paola Sbardella, Manuela Stefanelli, Giuseppe Pomarico, Cecilia Bombelli, Francesca Ceccacci, Roberto Paolesse, Mariano Venanzi, Donato Monti

**Affiliations:** 1 Department of Chemical Science and Technologies, University of Rome Tor Vergata, 00133 Rome, Italy; paola_sbardella@goodyear.com (P.S.); roberto.paolesse@uniroma2.it (R.P.); venanzi@uniroma2.it (M.V.); 2 Department of Chemistry, Sapienza University of Rome, 00185 Rome, Italy; giuseppe.pomarico@uniroma1.it (G.P.); donato.monti@uniroma1.it (D.M.); 3 Institute for Biological Systems (ISB), National Research Council of Italy (CNR), Secondary Office of Rome-Reaction Mechanisms c/o Department of Chemistry, Sapienza University of Rome, Piazzale A. Moro 5, 00185 Rome, Italy; cecilia.bombelli@cnr.it (C.B.); francesca.ceccacci@cnr.it (F.C.)

**Keywords:** surfactants, porphyrins, aggregation, micelles, chirality, circular dichroism, proline

## Abstract

The chirality transfer from chiral domains to achiral molecules is an important theoretical and applicative issue. In this work, we have investigated the interaction between two anionic chiral surfactants bearing a proline residue as hydrophilic head and the cationic, achiral porphyrin Zn(II) [5-{4-(3-trimethylammonium)propyloxyphenyl}-10,15,20-triphenylporphyrinyl]chloride to assess the effects of the structural variations in both units on the chirality transfer efficiency and amplification. We showed that the efficiency of transferring molecular information depends on the surfactant’s features, namely the chiral configuration of the polar head, the length of the aliphatic chain, and the aggregation state. At the same time, the presence of a coordinated metal and the peripheral charged group on the porphyrin macrocycle are key factors. In detail, the study of the hetero-aggregates formed at a surfactant concentration below the critical micellar concentration (cmc) indicates that the chirality depends on the synergy of hydrophobic effect, coordination interaction, and electrostatic forces. If the surfactant concentration is higher than the cmc, at a low concentration, porphyrins are included in micelles as monomers. Under these conditions, no chirality transfer is evident. When the porphyrin is in excess with respect to the micelles, an efficient asymmetry induction is again observed, transmitted from the chiral polar head to the porphyrin oligomers included in the micelle, through the polar heads and the hydrocarbon chains of the surfactants.

## 1. Introduction

Nature is driven by asymmetry. Under many other circumstances, nature prefers “one option out of two” in its assumed shapes, generating chiral species at a macroscopic level, as in the shell of a Nautilus linnaeus or the floret on the sunflower head. Similarly, the phenomenon is observed at the molecular scale in the specific configuration of DNA (the most common B-DNA is right-handed) or in the building blocks of life, such as amino acids or carbohydrates (having L- and D- configurations, respectively). It has been suggested that biological chirality is a consequence of the atom’s “homochirality”, arising from electroweak force parity violation [1].

The presence in nature of chiral molecules with a specific spatial arrangement of atoms has outstanding effects in many fields: from the development of synthetic techniques able of controlling or modulating the stereochemistry of the product [2], to the investigation of the specific optical, electrical, and magnetic properties of enantiomers/diastereomers [3], to the detection of chiral metabolites or pollutants [4].

Interest in molecules with a distinct stereochemistry has triggered the development of enantioselective synthetic techniques and has promoted improvement in chiral discrimination. Both stereospecific synthesis and enantiomer recognition resemble enzyme machineries, where the active site is a pocket structured to perform chiral recognition.

As an alternative to synthetic methods, a supramolecular approach based on non-covalent interactions driving spontaneous self-assembly has been pursued to generate chiral architectures. Understanding the interactions of chiral species and the transmission of their chiral information across various length scales and through space is challenging and is of great interest in multiple fields, including biology and materials science. Numerous studies, particularly those focused on supramolecular polymers, have identified the “sergeants-and-soldiers” principle and the “majority rules” effect as key phenomena that enhance chirality in higher-order assemblies [5,6,7]. These effects arise from the interaction of achiral monomers with a small number of chiral ones, or from imbalances in enantiomeric excess, which ultimately influence the ‘global’ chirality of the final structure.

Although significant progress has been made in understanding the mechanisms of chirality transfer and amplification, this topic remains a hot issue today, given the potential it offers in the development of supramolecular nanomaterials [5,7].

Among the possible systems to study, micelles have generated significant interest.

They are nanosized structures formed through a bottom-up approach, driven by the hydrophobic effect that organises surfactant building blocks [8]. Their oil-in-water architecture features a hydrophobic lumen that solubilizes apolar or slightly polar compounds, allowing for higher local concentrations that enhance chemical processes. Unlike many other nanoparticles with a more complex morphological organisation, micelles are structurally simple and can form supramolecular structures simply by mixing under the right conditions.

Micelles can act as tiny environments for catalytic reactions, with the reaction’s location influenced by the size, shape, polarity, and functional groups of the substrates and catalysts [9]. Chiral micelles can be formed from units that have at least one stereogenic centre or by assembling achiral structures that gain asymmetry once the final structure is complete [10]. Many examples of stereoselective catalysis in micellar media use achiral surfactants, but chiral surfactants can create chiral micelles that promote stereoselectivity even with achiral catalysts. The synergy between the chirality of both the catalyst and micelle offers new opportunities in asymmetric catalysis [9].

Importantly, the chirality of micelles can be further transferred and amplified to a supramolecular system obtained by interaction with achiral compounds, such as porphyrinoids [11,12,13,14,15,16,17]. Furthermore, fine-tuning the porphyrin molecular structure and the reaction conditions allows for the chiroptical properties of the final assemblies to be controlled [18,19,20,21,22]. Tetraarylporphyrins are synthetic molecules closely related to their natural counterparts [23]. Due to their facile synthesis [24], noticeable optical properties [25], and the ability to act as a chelating system towards many ions [26], porphyrinoid-based structures have been studied in many fields, such as materials science [27,28,29,30,31] or as biomimetic systems [32,33]. Extended electronic delocalisation confers a net hydrophobic character to porphyrins. However, the incorporation of a single ionic group turns these macrocycles into amphiphilic compounds, thus promoting specific interaction with surfactants. Since the composition of micelles is very similar to phospholipid bilayers, a supramolecular system combining micelle and porphyrin can be used to mimic the macrocycle interaction with biological membranes, as it occurs for the cytochrome P450 system, or to gain deeper insight into the mechanism of PDT in related diseases [34,35,36,37].

In this context, this work investigates the chirality transfer from chiral surfactants to an amphiphilic, achiral porphyrin, exploring different surfactant concentrations, i.e., above and below its cmc. Indeed, the aggregation state of the interacting molecules to be incorporated in organised microenvironments is a crucial aspect to consider.

**Figure 1:**
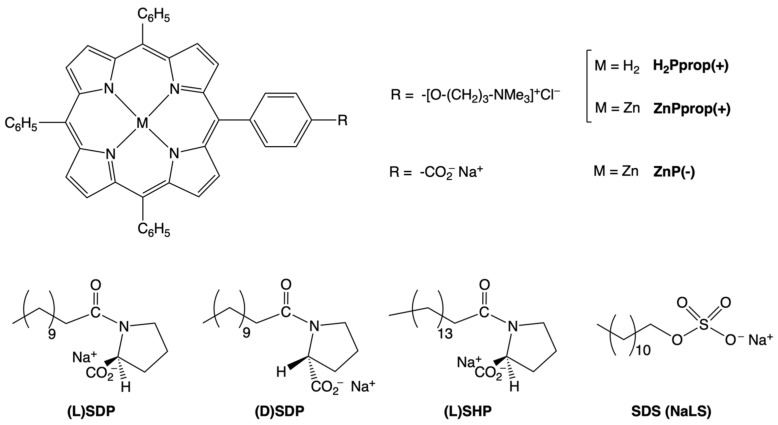
Porphyrin derivatives and anionic surfactants under investigation in the present work.

The porphyrin derivatives and the surfactants used in the present work are reported in Figure 1. The structural variations within the same class of molecules were carefully selected to gain as much information as possible on the interactions between the chromophores and the surfactants. Herein, we demonstrate that the alkyl chains of the surfactant molecules play a prominent role in determining the macrocycle inclusion and the efficiency of chirality transfer, highlighting that the hydrophobic effect is the main driving force for the process. The results are further compared to those obtained in the case of an achiral surfactant. In addition, metal coordination and electrostatic forces are crucial for the supramolecular chirogenesis process, which was investigated in depth by using different spectroscopic techniques (UV-Vis absorption, circular dichroism, fluorescence, and resonance light scattering).

The reported results are significant across multiple fields, including the development of new surfactant-based soft materials [38,39,40] and drug delivery platforms [41,42,43,44], as well as in stereoselective organocatalysis [9], involving photo- and electrochemical processes [45,46].

## 2. Results

The porphyrin investigated in this work is a Zn-tetraphenylporphyrin derivative functionalised by an alkylammonium (chloride) moiety, **ZnPprop(+)** [47], possessing an amphiphilic character which is well suited to the construction of supramolecular systems in aqueous micellar environments. The surfactants are anionic (as sodium salt) alkyl derivatives of (L)- or (D)-proline, possessing a different chain length (i.e., dodecyl and hexadecyl groups, SDP and SHP, respectively, in Figure 1) [48,49]. The SDS surfactant, characterised by a similar structure and a comparable cmc value to SDP, was also used as an achiral reference for comparison. These compounds were selected to evaluate the effect of different structural parameters on the chirality transfer efficiency: the alkyl chain length, the surfactant aggregative state, and the chiral configuration of the polar head. Table S1 reports the cmc values and the aggregation numbers for the three selected surfactants [49], which are fundamental parameters to consider in designing and commenting on the reported experiments.

Before going into detail about the results, it is necessary to define the R and R’ parameters that will often be recalled in the text during the discussion.

We define the R and R’ ratios, depending on the surfactant concentration, as follows:

R = [porphyrin]/[surfactant] when [surfactant] < cmc

R’ = [porphyrin]/[micelles] when [surfactant] > cmc

Micellar concentration was calculated using the following equation:

$$\left[micelles\right]=\frac{\left[total\;surfactant\right]-cmc}{n}$$

where *n* is the number of surfactant molecules forming the micelles. It is worth pointing out that the different cmc values of the two tested chiral surfactants allowed complementary investigations to be performed. Indeed, even if with (L)SDP it was not possible to study the porphyrin aggregation in micelles (the cmc value is too high), it allows for an easy variation in the R ratio. On the contrary, (L)SHP has proved particularly useful for studying the chirality induction on the micellised aggregates, making it easy to vary the R’ value. However, its use in concentrations below the cmc (C < 10^−6^ M) limited the investigation to solutions with R << 1.

As also reported in the literature [12,50], porphyrin systems in aqueous solutions of surfactants can lead to multiple homo- and hetero-aggregation processes, critically depending on both the porphyrin and surfactant concentrations. Moreover, in the case of the hetero-aggregation, it is essential to distinguish between porphyrin–surfactant hetero-aggregates formed at surfactant concentration below the cmc and the solubilization of porphyrin monomers and porphyrin homo-aggregates into the micellar phase, which represent competitive processes depending on the monomer/aggregate equilibrium in solution and on the different binding affinity of monomers and aggregates to the micellar phase. All of these systems can be obtained and characterised, depending on the experimental conditions used.

In the following, the most significant results on the chirality transfer from surfactants to achiral porphyrin platforms are presented, highlighting how the structural features of the surfactants, as well as their concentration, affect the efficiency of the process, due to the interplay between macrocycle–surfactant and macrocycle–macrocycle interactions. The use of other porphyrin derivatives, lacking of Zn ion coordinated or negatively charged onto the peripheral position, **H_2_Pprop(+)** and **ZnP(−)**, respectively, gave essential insights for the assessment of the driving interactions that rule the formation of supramolecular homo- and hetero-aggregates.

### 2.1. Chirality Transfer from SDP and SHP Chiral Surfactants

#### 2.1.1. The Effect of the Chiral Configuration of the Surfactant Polar Head: Studies on (L)/(D)SDP

**Figure 2:**
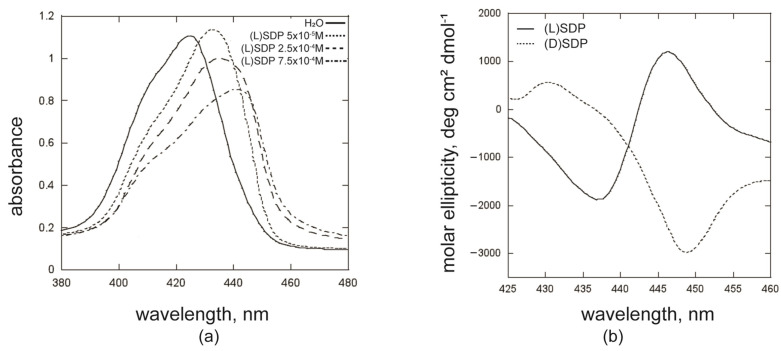
(**a**) UV-Vis spectra of **ZnPprop(+)** in different aggregation conditions and (**b**) CD spectra of porphyrin/surfactant hetero-aggregates formed by (L)/(D)SDP enantiomers. ([surfactant] = 2.5 × 10^−4^ M; R = 0.1). In (**a**,**b**), [**ZnPprop(+)**] is 2.5 × 10^−5^ M.

Tetraarylporphyrins are essentially hydrophobic and self-assemble in aqueous environments. In pure water, the hydrophobic effect drives the macrocycle’s aggregation, overwhelming other potential interactions that would induce a specific molecular recognition, leading to well-organised and morphologically defined suprastructures. This phenomenon also occurs when **ZnPprop(+)** is dissolved in water, as shown in the UV-Vis spectrum reported in Figure 2a (full line); the broad Soret band with an absorption maximum at 425 nm and a shoulder at ca. 410 nm is a typical indicator of the coexistence of an ensemble of porphyrin homo-aggregates with different morphologies. When aggregation is carried out in the presence of (L)SDP surfactant at concentrations below the cmc value, UV-Vis spectral features are markedly red-shifted, with the Soret band maxima falling in the 435–450 nm region, depending on the surfactant concentration (Figure 2a, dotted lines). This can be ascribed to a difference in ratio between the homo-aggregated and the hetero-aggregated species, the latter increasingly structured/arranged in J-type systems as the surfactant concentration boosts.

Regarding the chirality of these systems, the homo-aggregated species are CD-silent, as expected for assemblies from achiral macrocycles. Using the SDP enantiomers allows for the role of the surfactant polar head (i.e., the chiral effector) on the chirality transfer process to be evaluated. CD spectroscopy measurements at both (L)- and (D)SDP concentrations of 2.5 × 10^−4^ M (< cmc) and [**ZnPprop(+)**] = 2.5 × 10^−5^ M (R = 0.1) revealed that porphyrin–surfactant hetero-aggregates express chirality at the supramolecular level, evidencing that chirality is efficiently transferred from surfactant molecules to porphyrin macrocycles. Remarkably, this process is highly stereospecific, being strictly dependent on the chiral configuration of the surfactant polar head [51]. As shown in Figure 2b (full line), when (L)SDP is used, a positive, bisignated (+/−) CD spectrum is observed for the hetero-aggregates. At the same time, the combination of the cationic porphyrin with the (D) surfactant enantiomer gives rise to a reasonably mirrored, negative pattern (−/+) (Figure 2b, dotted line). Finally, hetero-aggregated species are also formed when the SDS surfactant is used, but with silent CD spectra, as expected for achiral interacting components in the aggregation process. Regarding the stoichiometry of the porphyrin/(L)SDP system, the Job plot analysis (Figure S1) afforded a 1:1 ratio between the two components, which corresponds to a neutral net charge, as also shown by previous studies on analogous systems [51,52,53].

#### 2.1.2. The Effect of the Surfactant Alkyl Chain Length: (L)SDP Versus (L)SHP

**Figure 3:**
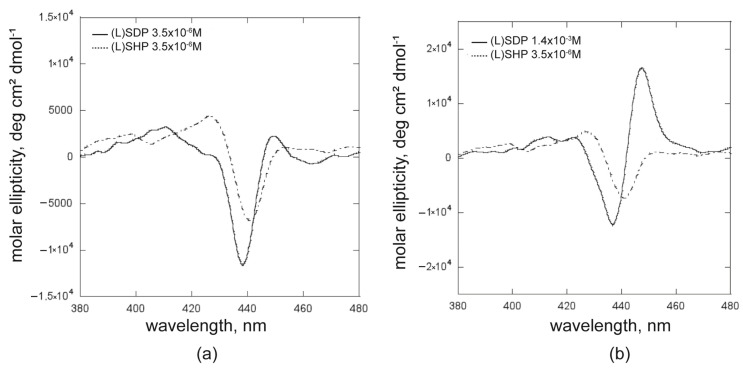
CD spectra of premicellar porphyrin/surfactant hetero-aggregates formed by (L)SDP (full lines) and (L)SHP (dotted lines): (**a**) at the same surfactant concentration and (**b**) in comparable surfactant aggregative state; ([**ZnPprop(+)**] = 1.0 × 10^−5^ M).

The first investigations were performed at concentrations below the cmc values for both surfactants. Different experimental conditions were also investigated to compare the effect of the surfactant alkyl chain length on the heteroaggregates’ chiroptical features. Figure 3 shows the recorded CD spectra for the experiments carried out at a 1.0 × 10^−5^ M porphyrin concentration, in the case of (*i*) [surfactant] = 3.5 × 10^−6^ M, which is largely under the cmc value for both surfactants, and (*ii*) [(L)SDP] = 3.5 × 10^−6^ M and [(L)SHP] = 1.4 × 10^−3^ M. These latter conditions enable comparisons at surfactant concentrations of one order of magnitude lower than the cmc value for both. In the first case, it is essential to underline that although the experiments were carried out at the same R value, the remarkable difference in cmc would cause the concentration of surfactant premicellar species to differ significantly in the solution. As clearly shown in Figure 3, all obtained hetero-aggregates are chiral, even if an evident influence of the surfactant structure on the overall asymmetry can be detected. Notably, when the (L)SHP is more organised than the (L)SDP counterpart (i.e., concentration closest to the cmc value), the chirality transfer from the (L)SHP is less efficient compared with that observed for the (L)SDP/**ZnPprop(+)** hetero-aggregates. This outcome indicates that the macrocycles’ arrangement, driven by the chiral configuration of the surfactant polar head, is strictly influenced by the overall molecular structure of the entities with which they associate. The shortest SDP’s alkyl chain favours a tighter structural macrocycle arrangement, where chromophores are more likely to interact closely, as evidenced by the emergence of the bisignated band in Figure 3b (full line), indicating a strong exciton coupling between the porphyrin macrocycles. This hypothesis was supported by the fluorescence and resonance light scattering studies, later discussed in the text. Nevertheless, the dichroic signals of the hetero-aggregated systems maintain the same sign, notably indicating that the specificity of the chirality transfer does not depend on the state of organisation of the surfactants.

**Figure 4:**
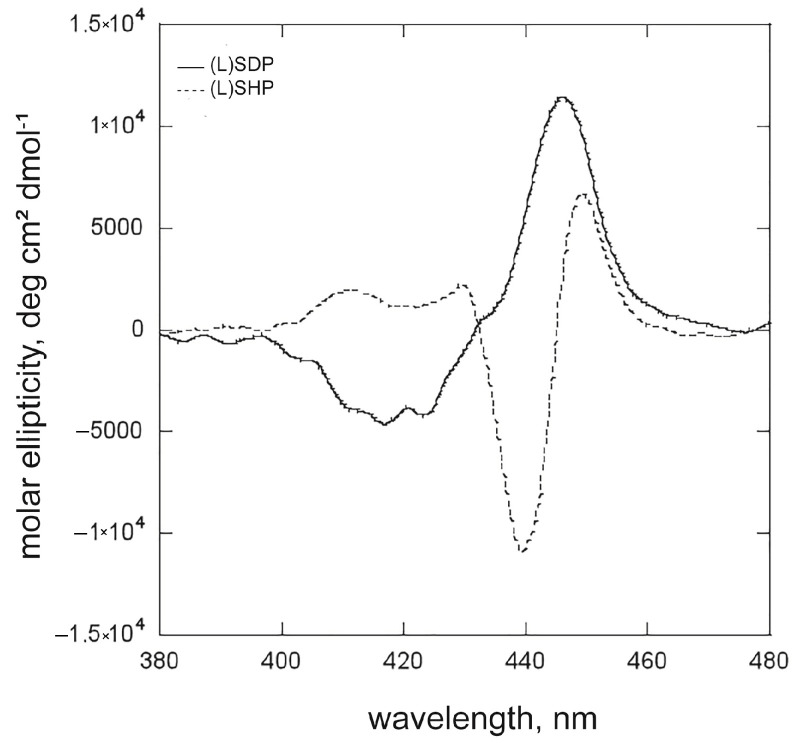
CD spectra of premicellar porphyrin/surfactant hetero-aggregates formed by (L)SDP at concentration below the cmc (full line) and by (L)SHP at concentration higher than cmc (dotted line); [**ZnPprop(+)**] = 2.5 × 10^−5^ M.

A further experiment consisted of using the same surfactant concentrations (5.0 × 10^−5^ M), which represent two remarkably different conditions, since this value is below cmc for (L)SDP, while for the (L)SHP, it is higher (for (L)SDP, R = 0.5; for (L)SHP, R’ = 80.8). At this value, the concentrations of (L)SHP surfactant in the premicellar and micellized phases are comparable; thus, the distribution of the porphyrin macrocycles in these two systems could be expected. Presumably, two distinctive bands in the corresponding CD spectrum should appear. The CD spectra for the (L)SHP reported in Figure 3 and Figure 4 showed substantial differences in the chiroptical features of the hetero-aggregated species, strictly depending on the surfactant aggregative state. This evidence prompted us to further investigate the dependence of the aggregation and inclusion in the micellar phase on the SHP concentration.

#### 2.1.3. The Effect of the Surfactant Concentration on Porphyrin Aggregation: The Case of (L)SHP

**Figure 5:**
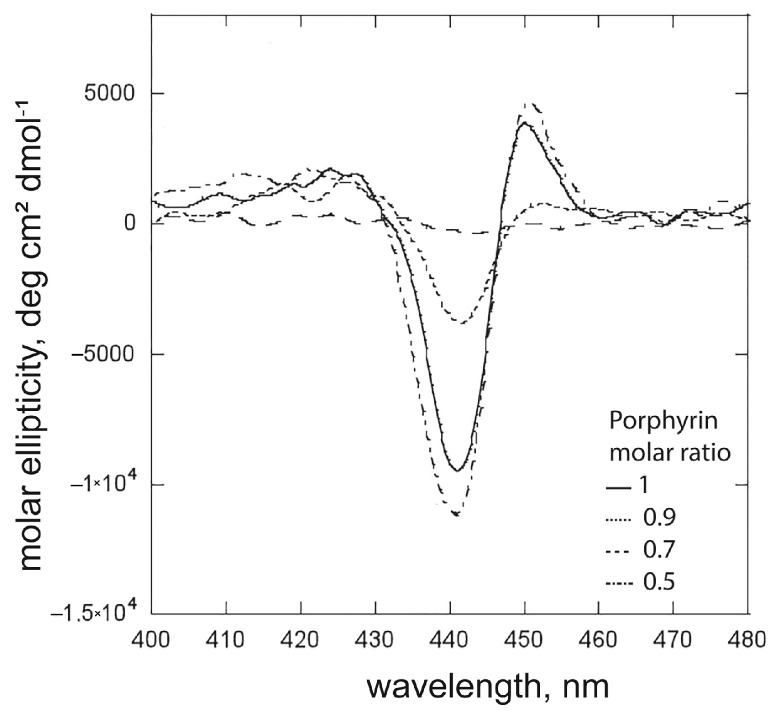
CD spectra of the solutions of the Job plot analysis, where [(L)SHP] is below the cmc.

Job plot analysis assessed the porphyrin/surfactant stoichiometry in the hetero-aggregated species (Figure S2a). As just observed for the (L)SDP, the maximum of the graph is located at the χ_ZnPprop(+)_ = 0.5, confirming that the favoured stoichiometry for the heteroaggregates obtained at concentrations below the cmc is 1:1, where the higher structural order can be conferred from the neutralisation of the charges of the molecular components. (L)SHP concentrations varied in the 0–4.5 × 10^−5^ M molar range. As reported in Figure S2b, the increase in [(L)SHP] causes the red shift in the Soret band from 424 to 434 nm, the latter corresponding to the porphyrin molar fraction of 0.5, where the surfactant concentration is proximal to the cmc. It is interesting to note that in the solutions where the micelle concentration is significant (χ_ZnPprop(+)_ = 0.3 and 0.1), the UV-Vis maximum shifts to 431 nm. As discussed below, this can be ascribable to the inclusion of porphyrin homo-aggregates inclusion micelles. As reported in Figure 5, CD spectra showed an increase in intensity with the (L)SHP concentrations, where the isodichroic point indicates the formation of aggregates with a similar morphology.

**Figure 6:**
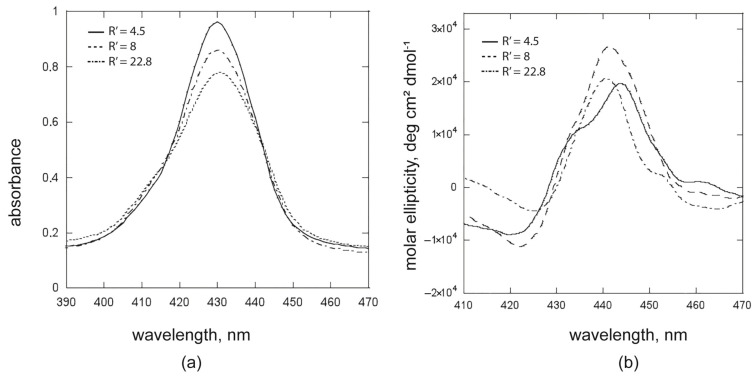
(**a**) UV-Vis spectra and (**b**) the corresponding CD spectra of solutions with [(L)SHP] = 1.2 × 10^−4^ M (R’ = 4.5); 7.6 × 10^−5^ M (R’ = 8) and 4.2 × 10^−6^ M (R’ = 22.8); [**ZnPprop(+)**] = 5 × 10^−6^ M).

When (L)SHP is employed at concentrations slightly higher than cmc (i.e., [**ZnPprop(+)**] = 5.0 × 10^−6^ M; [surfactant] = 1.2 × 10^−4^ M, 7.6 × 10^−5^ M, or 4.2 × 10^−5^ M, with R’ = 4.5, 8, and 22.8, respectively; UV-Vis spectra in Figure 6a) where the micellar phase is prevalent, the chirality transfer still occurs, but differences in the chiroptical features compared with the hetero-aggregated systems previously discussed can be highlighted. In particular, the crossover point in the CD spectra is located at 430 nm, thus 10 nm blue-shifted compared to the hetero-aggregated species; this effect confirms that the aggregates are surrounded by a less polar environment, which is the one offered by the micellar core (Figure 6b).

It is interesting to note that in all the tested solutions, micelle concentrations are consistently low ([micelles] = 1.0 × 10^−6^ M for R’ = 4.5; [micelles] = 5.0 × 10^−7^ M for R’ = 8; [micelles] = 1.0 × 10^−7^ M for R’ = 22.8). Nevertheless, the spectral variations suggest that the inclusion of homo-aggregates inside the micelle is preferred over the hetero-aggregation or even homo-aggregation outside the micelle.

A careful inspection of the CD spectra in Figure 6b reveals that, going from R’ 4.5 to 22.8, the band for the first Cotton effect at 440 nm turns into two overlapped bands at 440 and 430 nm, suggesting the co-presence of two families of aggregates within the micelle. This scenario was further highlighted by studying the solution with R’ = 9.2, where [**ZnPprop(+)**] = 2.5 × 10^−6^ M and [(L)SHP] = 2.5 × 10^−4^ M. Indeed, the early CD spectrum (Figure S3, curve a) evolves within 2 h in a double band, indicating the presence of two aggregated species of different morphology (Figure S3, curve b). In the case of the solution with R’ = 22.8, these bands are immediately observed because of the higher porphyrin concentration (Figure 6b). Fluorescence studies further shed light on the coexistence of different aggregates within the micelle, excluding the fact that the two bands are associated with an equilibrium between the porphyrin monomers and the aggregates (vide infra).

### 2.2. Fluorescence and Resonance Light Scattering Studies for the Surfactant–Porphyrin Systems

The structural differences among all the investigated hetero-aggregated systems were highlighted by fluorescence and resonance light scattering analyses. The following targeted solutions were studied: 5 × 10^−6^ M of **ZnPprop(+)** in 5.0 × 10^−6^ M (R = 1) and 0.1 M (L)SDP, and 5.0 × 10^−6^ M (R = 1), 7.6 × 10^−5^ M (R’ = 8), 3.8 × 10^−5^ M (R’ = 30), and 0.1 M (L)SHP (Table 1, entries 1–6).

For comparison, two further solutions were investigated: [**ZnPprop(+)**] dissolved in water, and in a 50/50 (*v*/*v*) ethanol/water solution, where the macrocycles are dissolved as monomers in a medium of similar polarity to the aqueous surfactant solutions (entries 7–8 in Table 1).

Figure S4 shows that the fluorescence spectrum in water of **ZnPprop(+)**, characterised by two emission bands located at λ_max_ = 603 and 639 nm, revealed a strong quenching of fluorescence emission intensity compared with the ethanol/water solution.

The hetero-aggregation of **ZnPprop(+)** with (L)SDP and (L)SHP surfactants modifies the wavelength and the intensity of the porphyrin emission bands for all the solutions investigated (Table 1).

**Figure 7:**
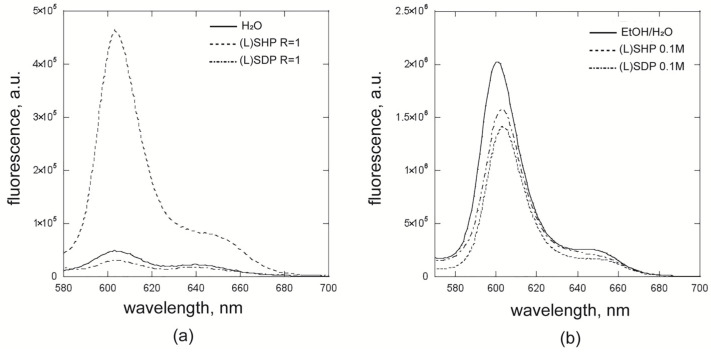
(**a**) Fluorescence spectra of three different systems: homo-aggregates of **ZnPprop(+)** in water (entry 7, Table 1) (full line) and hetero-aggregates formed in (L)SDP and (L)SHP solutions (entries 2 and 4, Table 1) (dotted line and dot-and-dash line, respectively). (**b**) Fluorescence spectra of porphyrin monomers in EtOH/H_2_O (entry 8, Table 1) (full line) and (L)SDP and (L)SHP micelles, (entries 1 and 3, Table 1) (dotted line and dot-and-dash line, respectively).

Table 1 shows that the second emission band is primarily affected by the surfactant and the porphyrin aggregative state (i.e., not directly linked to the solvent polarity). When porphyrins are in the monomeric form (entries 1, 3, and 8), the second emission band is placed at 645 nm, while in the case of aggregated systems, this band is below 640 nm, with some slight bathochromic shifts of ca. 2–3 nm. A substantial difference between the hetero-aggregates formed by the two surfactants is also visible (entries 2 and 4 in Table 1). However, the most crucial aspect to consider is the variation in fluorescence intensity in the different conditions, which is an indicative sign regarding the aggregative state of the porphyrin. CD studies revealed a marked difference between the hetero-aggregated species from (L)SDP and (L)SHP. Fluorescence measurements further highlighted this difference in terms of quantum yield. Indeed, the reported spectra (Figure 7a) showed the higher fluorescence quenching found for the (L)SDP surfactant, indicative of a stronger interaction between the porphyrin platforms. Furthermore, it is worth noting that the fluorescence intensity for the (L)SHP/porphyrin heteroaggregates is higher than that of the porphyrin homo-aggregates in water (Figure 7a). This evidence can be explained by invoking a looser geometry for the heteroaggregates. A clear difference in the position of the second band maximum is also detectable, being 639, 640, and 647 nm for water, (L)SDP, and (L)SHP, respectively.

The comparison of the two surfactants shows that, with the environmental polarity being equal, this fluorescence band is strictly influenced by the chromophore aggregative conditions. A further comparison between the porphyrins included as monomers and those of the monomers in EtOH/H_2_O medium is reported in Figure 7b. A slight fluorescence quenching for the porphyrin monomers in micellar systems (entries 1 and 3, Table 1), if compared with the monomers in the hydroalcoholic mixture, can be reasonably explained by the more rigid environment exerted by the micelles.

The fluorescence spectra for three solutions of (L)SHP, having R = 1, R’ = 8, and R’ = 30 (entries 4, 5, and 6, Table 1) are reported in Figure S5. In all the aggregative conditions, a decrease in the fluorescence intensity with respect to the porphyrin monomer is observed, being more pronounced for the solutions where the surfactant is used at concentrations higher than the cmc. This confirms that hetero-aggregates are more loosely packed than those observed in micelles. Furthermore, the second band in the fluorescence spectrum of the aggregates within the surfactant (λ = 636 nm) does not correspond to that of the monomer included and differs even from that of the heteroaggregates (λ = 647 nm). This also proves that in the case of the solution with R’ = 30, which is similar to that of R’ = 28, shown above, no free monomers are present.

**Figure 8:**
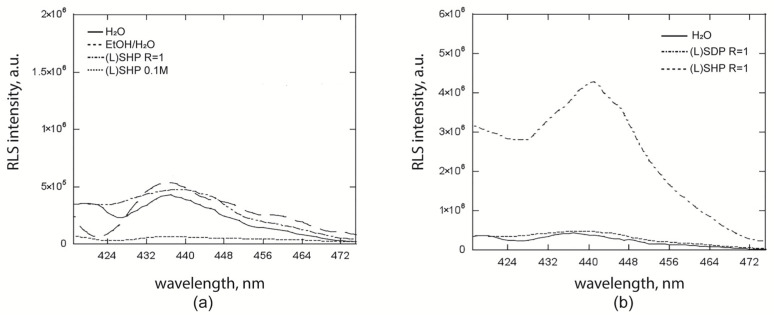
(**a**) RLS spectra of **ZnPprop(+)** at different aggregative conditions: in water, in EtOH/H_2_O solution, in the hetero-aggregated structures with (L)SHP (R = 1), and included as monomer in micelle ([(L)SHP] = 0.1 M). (**b**) RLS spectra of homoaggregates of **ZnPprop(+)** in water (full line) and the heteroaggregates formed with (L)SDP and (L)SHP for R = 1 (dotted line and dot-and-dash line, respectively) ([**ZnPprop(+)**] = 5 × 10^−6^ M).

Resonance light scattering (RLS) studies gave further evidence of the structural differences observed in the porphyrin/surfactant systems, since this technique allows electronically coupled chromophore arrays to be studied [54,55]. As expected, the RLS spectra for the solutions where porphyrins are monomers (entries 1 and 8, Table 1) have a negligible intensity (lines b and d, Figure S6). Quite unexpectedly, the intensity is very low also for the **ZnPprop(+)** aqueous solution, evidencing that the macrocycles are in weak electronic conjugation (line a, Figure S6). Finally, RLS signal intensity was higher in the case of hetero-aggregates formed in (L)SDP (R = 1) solution compared to those in (L)SHP or water (Figure 8b). From the same comparison made for the (L)SHP solutions (Figure 8a), it is possible to note that the hetero-aggregates formed with this surfactant are characterised by a weaker inter-macrocycles interaction, confirming what is shown by CD and fluorescence measurements (i.e., looser hetero-aggregates for the (L)SHP/porphyrin systems, at surfactant concentration below the cmc). The RLS technique also supported the fact that increasing the R’-values caused porphyrin aggregation in micelles (Figure S7); the low intensity of the RLS signal suggests the presence of porphyrin oligomers, which consist of a limited number of chromophores exhibiting strong electronic conjugation, as evidenced by CD experiments. A final comparison is made regarding the hetero-aggregates formed by the porphyrin/surfactant systems below the surfactant’s cmc value (Figure 8b). The hetero-aggregates based on (L)SDP exhibit a higher RLS intensity compared to those formed with (L)SHP. This indicates a greater number of macrocycles in close electronic communication, likely forming more extended structures than those encapsulated within a micelle. This finding further confirms that the SDP surfactant possesses structural characteristics that enhance the efficiency of chirality transfer compared to (L)SHP.

### 2.3. Evaluation of the Interactions Involved in the Supramolecular Chirogenesis. The Case of **H_2_Pprop(+)** and **ZnP(−)** Derivatives

The chemical versatility of porphyrins allows for the structural modification of the macrocycle to investigate the role of the possible interactions involved in supramolecular chirogenesis. For such a purpose, two porphyrin derivatives were selected, i.e., **H_2_Pprop(+)** and **ZnP(−)** (Figure 1), the former lacking the zinc ion coordinated to the porphyrin core and the latter having an anionic peripheral group (-CO_2_^−^). Such variations make it possible to assess the role of metal coordination and attractive electrostatic interactions in the molecular recognition between porphyrin and the chiral surfactants tested. Using the (L)SHP surfactant allows the investigation of these issues in different environments, depending on the surfactant’s aggregative state. That is why we considered chiral surfactant concentrations below and above the cmc.

#### 2.3.1. Studies on **H_2_Pprop(+)** and **ZnP(−)**, with [(L)SHP] < cmc

When aggregation was performed in the following conditions, i.e., [(L)SHP] = 10 μM (< cmc) and [porphyrin] = 25 μM (R = 2.5), both **H_2_Pprop(+)** and **ZnP(−)** gave achiral aggregated species detectable by UV-Vis spectroscopy, as demonstrated by the negligible CD signals recorded. These outcomes suggest that both the coordination of the carboxylate ion of the surfactant and the electrostatic interaction between the polar head of the surfactant and the quaternary ammonium group on the porphyrin are discriminating factors for the evolution of highly specific suprastructures. Given that the stoichiometry of [surfactant]/[porphyrin] is 1:1, a likely graphical representation of hetero-aggregated species organised in extended suprastructures is given in Figure S8. We can surmise that the first interaction between the two molecules in solution is essentially electrostatic; then, the species reorganise, and the polar head of the chiral vector is coordinated to the Zn atoms of two close porphyrins. At the same time, the alkyl chains are intercalated among the pendants bound to the macrocycles. It is reasonable to suppose that the first Zn ion coordinates the surfactant’s carboxylate while the close macrocycle’s Zn interacts electrostatically with the carboxyl group. In this way, observing the overall structure, all metal ions interact with the surfactant. These alternating interactions are repeated through space, giving suprastructures with different extensions depending on the structure of the surfactant itself, as previously demonstrated by fluorescence and RLS studies.

#### 2.3.2. Studies on **H_2_Pprop(+)** and **ZnP(−)**, with [(L)SHP] > cmc

**Figure 9:**
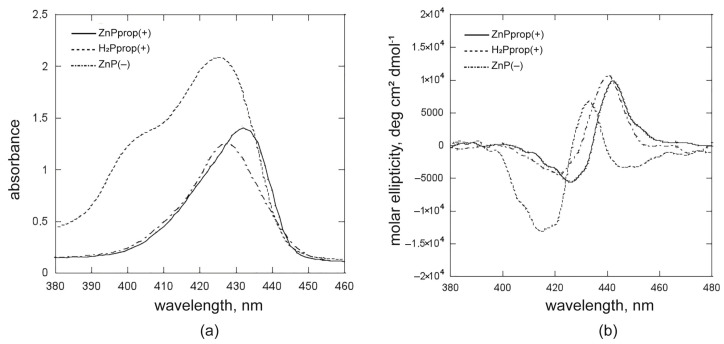
(**a**) UV-Vis and (**b**) the corresponding CD spectra of homoaggregates of **ZnPprop(+)**, **H_2_Pprop(+)** and **ZnP(−)** included in (L)SHP micelles; ([(L)SHP] = 2.5 × 10^−4^ M and [porphyrin] = 2.5 × 10^−5^ M (R’ = 9.2).

The aggregation process was optimised to enable micellar inclusion (R’ > 1) and to investigate how chirality is induced during this process. The experiments were carried out at the concentrations [(L)SHP] = 0.25 mM and [porphyrin] = 25 μM (R’ = 9.2) for both **H_2_Pprop(+)** and **ZnP(−)** derivatives. It is well-reported that the interaction between surfactants at concentrations higher than cmc and organic chromophores in aqueous solutions leads to the inclusion of the apolar portions of molecules in the hydrophobic region of the micelles [11]. The molecules that are highly soluble in water are generally not included, and those that are electrostatically repulsed from the micellar surface are also not included [12,56]. The UV-Vis and CD spectra in Figure 9 show that, in the investigated conditions, the inclusion in micelles in homo-aggregated forms occurs with chirality transfer even for the derivatives that did not form specific structures at surfactant concentration below the cmc. This demonstrates that the hydrophobic effect guiding the inclusion offers an asymmetric, organised domain crucial for forming chiral porphyrin aggregates. The investigated porphyrin derivatives have marked solvophobic features, as evidenced by their tendency to aggregate in water. For this reason, their incorporation into micelles is favored, even if it means overcoming electrostatic repulsion in the initial stages.

## 3. Materials and Methods

### 3.1. Synthesis

The porphyrin derivatives **H_2_Pprop(+)**, **ZnPprop(+),** and **ZnP(−)** were prepared as previously reported by our group [47,57]. The (L)/(D)–SDP and (L)SHP chiral surfactants were synthesised following procedures outlined in the literature [45,58]. SDS is from a commercial source, at the highest degree of purity available, and was used as received. The cmc and the aggregation number n values of the surfactants used were determined in previous papers and are reported in Table S1 [49]. The solvents are of spectroscopic grade and were used as received.

### 3.2. Determination of the Molar Extinction Coefficient of ZnPprop(+) in Micelles

The molar extinction coefficient of **ZnPprop(+)** in the micelles has been determined in (L)SDP 0.2 M, (L)SHP 0.2 M, and in EtOH/H_2_O 50% (*v*/*v*).

To a 2.5 mL of surfactant solution, several aliquots of a 2.2 × 10^−4^ M porphyrin solution in EtOH (7, 8, 10, 10, and 15 μL) were added, yielding a porphyrin concentration varying from 1.2 to 8.5 μM. Solutions were kept at 25 °C for 30 min, and then the UV-Vis spectra were acquired.

The R’ values are, in all cases, below 4 × 10^−3^, ensuring that the porphyrin macrocycle is included in micelles in the monomeric form and the applicability of the Lambert–Beer equation for estimating molar extinction coefficients. The porphyrin in EtOH displays the Soret band at 423 nm, which remains almost fixed when macrocycles are included in micelles. The slight variations in the Soret maxima in the cases of (L)SDP and (L)SHP (426 and 427 nm, respectively) can be attributed to the variation in the chemical environment of the chromophore. Furthermore, the ε value has been calculated in a water–ethanol mixture (1:1, *v*/*v*), which reasonably represents the polarity of the micellar environment, as evidenced from ^1^H-NMR studies of several solutes [43]. The calculated values are as follows: in 0.2 M (L)SDP, λ_max_ (nm), ε (M^−1^, cm^−1^): 426 (2.60 × 10^5^); in 0.2 M (L)SHP, λ_max_ (nm), ε (M^−1^, cm^−1^): 427 (3.16 × 10^5^); and in EtOH/H_2_O (1:1, *v*/*v*), λ_max_ (nm), ε (M^−1^, cm^−1^): 423 (2.80 × 10^5^).

### 3.3. Spectroscopic Studies

UV-Vis spectroscopic measurements were performed on a Varian Cary 100 (Varian, Inc., Palo Alto, CA, USA) at 298 K. CD spectra were obtained on a JASCO J-600 (JASCO, Tokyo, Japan), equipped with a thermostated cell holder at 298 K, and purged with ultra-pure nitrogen gas. Solutions suitable for the aggregation studies were prepared as follows: in an 8 mL glass vial, a proper volume of water was added, followed by an aliquot of a concentrated solution of a surfactant and an aliquot (5–25 μL) of an ethanolic solution of porphyrin. A 2.5 mL portion of the resulting solution was rapidly transferred to a quartz cuvette, and the relative UV-Vis spectra were acquired.

Fluorescence measurements were collected on a Fluoromax-4 (HORIBA Scientific, Piscataway, NJ, USA) spectrofluorometer, using Milli-Q^®^ water (Merck, Darmstadt, Germany). RLS studies were carried out using a Spex Fluorolog Spectrofluorophotometer (HORIBA Scientific, Piscataway, NJ, USA), and the spectra were acquired in the 350–500 nm range.

## 4. Conclusions

We have investigated the interaction between two chiral anionic surfactants and an achiral cationic zinc porphyrin. The chiral element—namely, the polar head of the surfactant—can transfer its chirality to porphyrin-based systems depending on the experimental conditions. By exploring a broad range of surfactant concentrations, we identified different types of assemblies, ranging from porphyrin–surfactant hetero-aggregates at surfactant concentrations below the cmc value, to porphyrins incorporated into micelles either as homo-aggregates (R’ > 1) or as monomers. As expected, in the latter case (monomeric porphyrins within micelles), no chirality transfer from the surfactant to the tetrapyrrolic macrocycle is observed.

In contrast, chiral systems are obtained in the other cases, where the chirogenesis arises from a combination of Coulombic and coordinative interactions, and hydrophobic effects, with their relative contributions depending on the surfactant-to-porphyrin ratio. When the surfactant is present as monomers (below the cmc), it interacts with the porphyrin to form hetero-aggregates, driven by electrostatic interactions between the tetraalkylammonium and carboxylate groups, as well as coordination of the surfactant’s polar head to the Zn centre of the porphyrin. The length of the surfactant’s alkyl chain influences both the chiroptical properties and the tightness of these hetero-aggregates, as clearly shown by fluorescence and resonance light scattering (RLS) studies.

When the surfactant concentration slightly exceeds the cmc, porphyrins are incorporated into micelles as aggregated species. This indicates that, in these highly organised systems, the surfactants can transfer their stereochemical information to the entire porphyrin aggregate. In this case, the chirality transfer is driven primarily by the hydrophobic effect, which overrides the structural features of the porphyrin derivatives, namely the charge of the peripheral groups and the presence of the coordinated Zn(II) ion.

## Figures and Tables

**Table 1:** Effect of the (L)SDP and (L)SHP surfactants in different aggregative conditions on the excitation and emission maxima of **ZnPprop(+)** (λ_ex_ 559 nm).

Entry	Solution	λ_max_ ^em^ (1), nm	λ_max_ ^em^ (2), nm
1	(L)SDP 0.1 M	603	645
2	(L)SDP R = 1	604	640
3	(L)SHP 0.1 M	603	645
4	(L)SHP R = 1	603	647
5	(L)SHP R’ = 8	604	636
6	(L)SHP R’ = 30	603	637
7	H_2_O	603	639
8	EtOH/H_2_O	601	645

## Data Availability

The original contributions presented in this study are included in the article/Supplementary Materials. Further inquiries can be directed to the corresponding author.

## Supplementary Materials

The following supporting information can be downloaded at https://www.mdpi.com/article/10.3390/ijms262311330/s1.

## Abbreviations

The following abbreviations are used in this manuscript:

cmc	Critical Micellar Concentration
SDP	Sodium Dodecyl Sulphate
SHP	Sodium Hexadecyl Sulphate
H_2_Pprop(+)	[5-{4-(3-trimethylammonium)propyloxyphenyl}-10,15,20-triphenylporphyrin]chloride
ZnPprop(+)	Zn(II) [5-{4-(3-trimethylammonium)propyloxyphenyl}-10,15,20-triphenylporphyrin]chloride
ZnP(−)	Zn(II) [5-{4-(carboxyphenyl}-10,15,20-triphenylporphyrinato
UV-Vis	Ultraviolet–Visible
CD	Circular Dichroism
RLS	Resonance Light Scattering
EtOH	Ethanol
