# Supplementary material for: Supramolecular Chirogenesis in Porphyrin-Based Systems: Chirality Transfer from Anionic Chiral Surfactants to Cationic, Achiral Porphyrins
Source: Int J Mol Sci. 2025 Nov 24;26(23):11330. doi: 10.3390/ijms262311330 (PMC12691959; doi:10.3390/ijms262311330)
Supplement: Supplementary file 1 [file ijms-26-11330-s001.zip › ijms-3961131-supplementary.pdf]

Supramolecular chirogenesis in porphyrin-based systems: chirality transfer from anionic chiral surfactants to cationic, achiral porphyrins

Paola Sbardella[1], Manuela Stefanelli[1], Giuseppe Pomarico[2], Cecilia Bombelli[3], Francesca Ceccacci[3], Roberto Paolesse[1], Mariano Venanzi[1], and Donato Monti[2,*]*

[1] Department of Chemical Science and Technologies, University of Rome Tor Vergata, 00133 Rome, Italy
[2] Department of Chemistry, Sapienza University of Rome, 00185 Rome, Italy
[3] Institute for Biological Systems (ISB), National Research Council of Italy (CNR), Secondary Office of Rome-Reaction Mechanisms c/o Department of Chemistry, Sapienza University of Rome, Piazzale A. Moro 5, 00185 Rome, Italy

* Correspondence: manuela.stefanelli@uniroma2.it Tel.:+390672594736; donato.monti@uniroma1.it Tel.:+390649913329

Supporting Information

Table S1: Aggregation parameters of the surfactants used, measured at 298 K.

Surfactant	*cmc*, M	Aggregation number, *n*
**(L)/(D)SDP**	$(9.6 \pm 0.8) \times 10^{-3}$	46 ± 2
**(L)SHP**	$(2.4 \pm 0.12) \times 10^{-5}$	84 ± 5
**SDS**	$(8.3 \pm 0.05) \times 10^{-3}$	67 ± 4

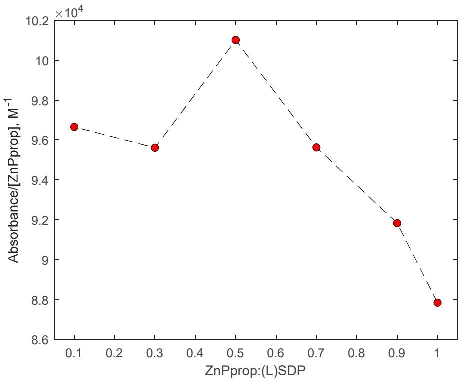

Figure S1: Job plot for the interaction of **ZnPprop(+)** with (L)SDP surfactant obtained by plotting the UV-Vis maximum absorbance vs. porphyrin molar fraction (at a total concentration of 5×10^{-5} M).

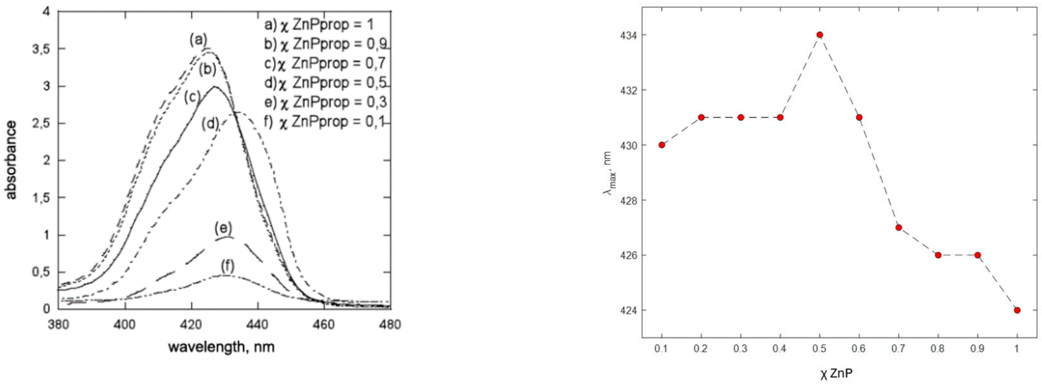

(a) **(b)**

Figure S2: (a) UV-Vis spectra of the solutions used for the Job plot analysis (on the left); (b) Job plot for the interaction of **ZnPprop(+)** with (L)SHP surfactant obtained by plotting the UV-Vis maximum wavelengths vs. porphyrin molar fraction (at a total concentration of 5×10^{-5} M) (on the right).

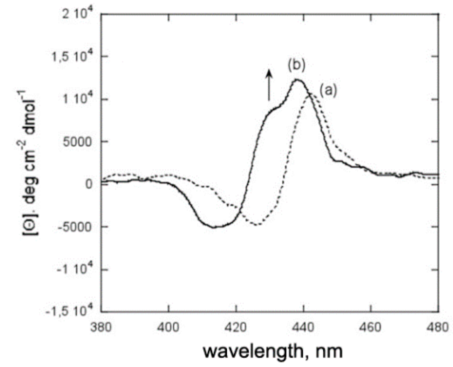

Figure S3: Evolution of porphyrin homo-aggregates in micelle of (L)SHP at t=0 (curve a) and at the equilibrium (curve b)(t=2h) [**ZnPprop(+)**] = 2.5 $\times 10^{-5}$ M; [(L)SHP]= 2.5 $\times 10^{-4}$ M; R'=9.2).

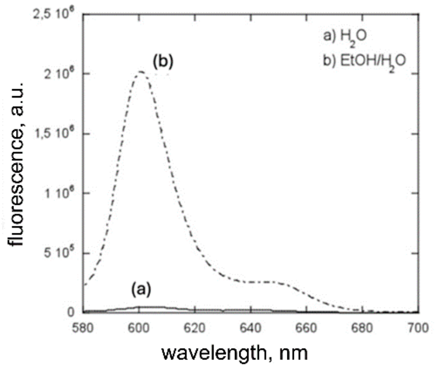

Figure S4: Fluorescence emission intensity of [**ZnPprop(+)**] = 2.5 $\times 10^{-5}$ M in H_2O (a) and EtOH/ H_2O (1/1, v/v) (b).

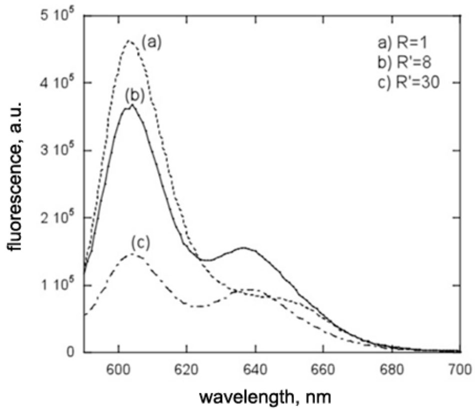

Figure S5. Fluorescence spectra of the hetero-aggregates at [(L)SHP] below the *cmc* (curve a) and porphyrin aggregates included in micelle (curves b and c), at different R' ratios. ([**ZnPprop**] = 5 ×10^{-6} M).

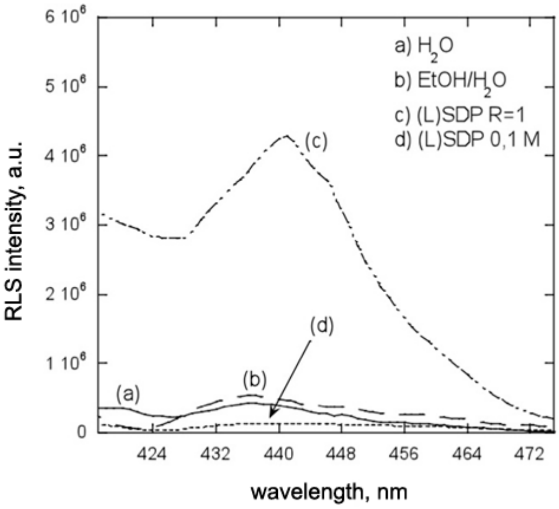

Figure S6: RLS spectra for the **ZnPprop(+)** in different aggregative conditions. ([**ZnPprop(+)**] = 5 ×10^{-6} M).

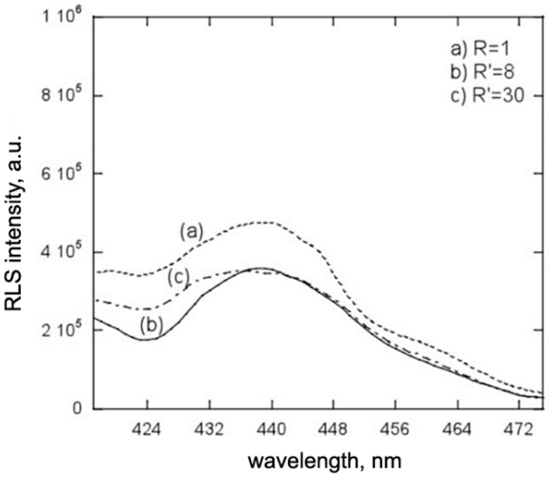

Figure S7. RLS spectra for the **ZnPprop(+)** in different aggregative conditions: a) heteroaggregates in (L)SHP at concentration below the *cmc*; b) porphyrin oligomers and c) porphyrin aggregates included in (L)SHP micelle ([**ZnPprop(+)**] = 5 $\times 10^{-6}$ M).

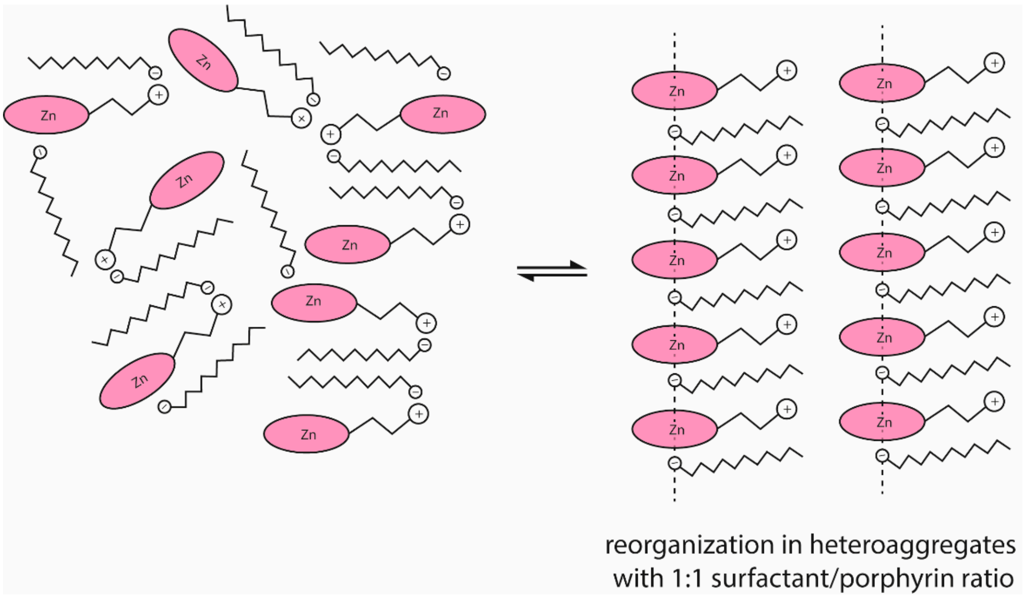

Figure S8. Illustration of the hypothesized evolution of the chiral porphyrin-surfactant hetero-aggregates.
